# Intraocular pressure and ocular biometric parameters changes in migraine

**DOI:** 10.1186/s12886-016-0258-5

**Published:** 2016-05-31

**Authors:** Yaran Koban, Hatice Kose Ozlece, Gorkem Bilgin, Mustafa Koc, Halil Huseyin Cagatay, Emre I. Durgunlu, Ayse Burcu

**Affiliations:** Department of Ophthalmology, Faculty of Medicine, University of Kafkas, Kars, Turkey; Department of Neurology, Edirne State Hospital, Edirne, Turkey; Department of Ophthalmology, Hacettepe University Beytepe Health Center, Ankara, Turkey; Ophthalmology Department, Ulucanlar Eye Training and Research Hospital, Ankara, Turkey; Kafkas University Medical School, Merkez, 36100 Kars, Turkey

**Keywords:** Anterior segment, Cornea, Headache, Miraine, Pain attack

## Abstract

**Background:**

The aim of this study was to assess the intraocular pressure and ocular biometric parameters in migraine patients during acute migraine attacks and compare them with painless period and healthy controls using a new optical biometer AL-Scan.

**Methods:**

In this prospective, case–control study, the axial length, corneal curvature radius, anterior chamber depth, central corneal thickness, and pupil size of 40 migraine patients during acute migraine attacks and painless period and 40 age- and sex-matched healthy subjects were measured using a *AL*-*Scan* optical biometer (Nidek Co., Gamagori, Japan). All patients underwent a complete ophthalmic examination before the measurements. IOP and biometer measurements were taken at the same time of day (10:00–12:00) in order to minimize the effects of diurnal variation.

**Results:**

There was not a statistically significant difference in intraocular pressure between the migraine patients during acute migraine attacks (15.07 mmHg), painless period (14.10 mmHg), and the controls (15,73 ± 0,81). Also, the ocular biometric parameters did not significantly vary during the acute migraine attacks.

**Conclusions:**

Further studies are needed to evaluate the etiopathologic relationship between intraocular pressure and ocular biometric parameters and acute migraine attack.

## Background

Migraine is a common disease that presents with a chronic, episodic, and disabling primary headache capable of causing significant dysfunction and coincides with neurological, gastrointestinal, and autonomic changes [1, 2]. Various vascular, neurovascular, hypoxic, cellular, hormonal, and genetic hypotheses have been debated in the migraine pathogenesis, yet the pathophysiology of migraine is still not fully understood [3, 4].

Cortical spreading depression (CSD), a wave of activity that propagates slowly across the brain surface, has been presumed since its original description in the 1940s to be the physiological substrate of the migraine aura [5]. Although this has been discussed for many decades as the underlying mechanism of the aura in migraine, it is still not clear how CSD is related to headache. In recent studies, CSD has been found to be associated with dramatic changes in the calibre of blood vessels and the electrophysiological activation of trigeminovascular neurons during a migraine attack [6]. Sensorial innervations of the eye are commonly originated from the trigeminal ganglion and are also supplied by the trigeminal nerve. Long and short ciliary nerve branches of the ophthalmic division of the trigeminal nerve innervate different parts of the eye, such as the iris, ciliary body, choroid, and trabecular meshwork [7].

Although several previous studies have reported decreased blood flow in the retina and optic nerve in migrainous patients and have demonstrated changes in the retinal and choroidal layer, no study has investigated the effect of migraine on changes in the intraocular pressure (IOP) and ocular biometric parameters in these patients [8, 9]. These parameters may provide valuable information for the risk assessment of glaucoma, calculation of intraocular lens power, monitoring of keratoconus, and the investigation of refractive disorders.

The aim of the present study was to investigate potential differences in the IOP, the axial length (AL), corneal curvature radius (CCR), anterior chamber depth (ACD), lens thickness (LT), central corneal thickness (CCT), corneal diameter (WtW), and pupil size between patients who have migraines during migraine attacks and the interictal period.

## Methods

The study adhered to the tenets of the Declaration of Helsinki. It was approved by the local ethics committee (*Kafkas University, Human Ethics Committee, Kars, Turkey)*, and written informed consent was obtained from all patients before they were recruited into the study.

A total of 40 patients with migraine with aura (32 females and 8 males with a similar mean age) were included in the study. Forty healthy volunteers (32 females and 8 males) were included in the control group. The eyes on the symptomatic side in migranous patients were included in the study. The diagnosis of migraine was made according to the International Classification of Headache Disorders, 3rd edition (beta version) (ICHD-3-beta) [10]. Patients were informed about the study and applications were provide during the attack period. Then, patients with acute migraine attack verified by a neurologist were referred to ophthalmology clinic immediately. All measurements were completed before the attack treatment and repeated at the same patients during pain- and attack-free periods. Patients’ detailed histories were recorded, and information about the frequency of migraine attacks, pain localization, age at onset, duration, and the presence of aura was recorded. The type of migraine was determined, and systemic comorbidities, presence of migraine in the family, and history of glaucoma were noted. Migraine subjects with other central nervous system diseases or abnormal magnetic resonance imaging (MRI) findings were excluded.

Volunteers who had full vision (1.0 with or without correction) and who had normal ocular findings were included in the study; patients with IOP > 21 mmHg, high spherical > ±3 dioptri or cylindrical > 2 dioptri refractive errors, and those who had history of uveitis, glaucoma, or ocular trauma previous eye surgery were excluded from the study. Moreover, a history of systemic disease such as hypertension or diabetes mellitus and any medication also resulted in patients being excluded from the study.

Full ophthalmologic evaluations including best-correct visual acuity, slit-lamp biomicroscopy, Goldmann applanation tonometry, gonioscopy with a three mirror contact lens, and fundoscopy were performed. AL, CCT, ACD, CCR, WtW, and pupil diameter (PD) were measured with a NIDEK AL-Scan® biometer during pain- and attack-free periods. The LT was obtained by A-scan ultrasound, and the IOP was measured by Goldmann aplanation tonometry (Model AT 900, Haag-Streit USA, Mason, OH, USA) during pain- and attack-free periods.

### Statistical analysis

For statistical analysis, SPSS 16.0 software for Windows (SPSS Inc, Chicago, IL) was used to analyze outcomes. *P* values < 0.05 were considered to be statistically significant. The normal distribution of the data was checked using the Kolmogorov-Smirnov test. The paired samples *t*-test was used for comparison of the parameters studied during pain- and attack-free periods in migraine patients. A comparison of anterior segment parameters between migraine patients and the control group was performed by an independent samples *t*-test.

## Results

In the migraine group, the average age was 34.5 years (±11.19); in the control group, the average age was 30.1 years (±14.15). There was no significant difference in age between the 2 groups (*p* = 0.116). The mean time required for migraine diagnosis was 88.8 ± 12.5 months (62–110). The mean attack frequency was 3.6/month in the migraine group.

The effects of the pain attacks on AL, CCT, ACD, CCR, WtW, PD, LT, and IOP in migraine patients are shown in Table 1. There were no significant differences in the mean AL, CCT, ACD, CCR, WtW, PD, LT, and IOP values between those taken during pain and the headache-free interval (*p* > 0.05).

**Table 1:** The effects of the pain attact on intraocular pressure and biometric parameters in migrain patients are shown

	Pain attact Mean ± S.D.	Attack-free periods Mean ± S.D.	*P*
IOP, mmHg	*15,08 ± 3,31*	13,94 ± 2,84	0,12
Range	(11–20)	(11–20)	
AL, mm	23,09 ± 1,2	*23,08 ± 1,2*	0,2
Pachymetry			
CCT, μm	536,16 ± 35,10	538,13 ± 35,42	0,16
ACD, mm	3,48 ± 0,24	3,40 ± 0,53	0,3
Keratometry readings			
*K*1, D	43,08 ± 1,75	43,11 ± 1,72	0,7
*K*2, D	44,33 ± 2,07	44,23 ± 2,03	0,3
LT, mm	4,52 ± 3,82	3,77 ± 0,34	0,3
PD, mm	5,94 ± 0,96	5,99 ± 0,92	0,4
WtW, mm	12,18 ± 0,43	12,20 ± 0,44	0,112

*IOP* intraoculer pressure, *AL* axial length, *CCT* central corneal thickness, *ACD* anterior chamber depth, *LT* lens thickness, *PD* pupil diameter, *WtW* corneal diameter

Furthermore, there were no significant differences in the mean AL, CCT, ACD, CCR, WtW, PD, LT, and IOP values between migraine patients and healthy participants (*p* > 0.05; see Tables 2 and 3).

**Table 2:** Intraocular pressure and biometric parameters in migraine patients during pain and healthy participants are shown

	Pain attact	Control	P
	Mean ± S.D.	Mean ± S.D.	
IOP, mmHg	*15,08 ± 3,31*	15,73 ± 0,81	0,5
Range	(11–20)	(11–20)	
AL, mm	23,09 ± 1,2	*23,3 ± 0,7*	0,4
Pachymetry			
CCT, μm	536,16 ± 35,10	533,20 ± 31,85	0,7
ACD, mm	3,48 ± 0,24	3,52 ± 0,25	0,4
Keratometry readings			
*K*1, D	43,08 ± 1,75	43,23 ± 1,52	0,7
*K*2, D	44,33 ± 2,07	44,19 ± 1,50	0,7
LT, mm	4,52 ± 3,82	3,76 ± 0,31	0,2
PD, mm	5,94 ± 0,96	5,97 ± 1,07	0,6
WtW, mm	12,18 ± 0,43		0,112

*IOP* intraoculer pressure, *AL* axial length, *CCT* central corneal thickness, *ACD* anterior chamber depth, *LT* lens thickness, *PD* pupil diameter, *WtW* corneal diameter

**Table 3:** Intraocular pressure and biometric parameters in migraine patients during interictal period and healthy participants are shown

	Attack-free periods Mean ± S.D.	Control Mean ± S.D.	P
IOP, mmHg	*14,11 ± 2,88*	15,81 ± 0,81	0,07
Range	(11–20)	(11–20)	
AL, mm	23,08 ± 1,2	*23,3 ± 0,7*	0,4
Pachymetry			0,5
CCT, μm	538,13 ± 35,42	533,20 ± 31,85	
ACD, mm	3,48 ± 0,23	3,52 ± 0,25	0,4
Keratometry readings			
*K*1, D	43,11 ± 1,72	43,23 ± 1,52	0,7
*K*2, D	44,23 ± 2,03	44,19 ± 1,50	0,9
LT, mm	4,47 ± 3,75	3,76 ± 0,31	0,3
PD, mm	5,99 ± 0,92	5,81 ± 1,07	0,4
WtW, mm	12,20 ± 0,44		0,112

*IOP* intraoculer pressure, *AL* axial length, *CCT* central corneal thickness, *ACD* anterior chamber depth, *LT* lens thickness, *PD* pupil diameter, *WtW* corneal diameter

## Discussion

The mechanisms underlying headache, aura, and photophobia in migraine are still not fully understood, although migraine is the most prevalent neurological disorder and much research has been done in that field. The most common known event in migraine pathogenesis is vascular dysregulation (vasospastic diathesis), and the neurovascular system is the most affected system in this pathology [4, 11, 12]. The new theory, CSD, is also found associated with dramatic changes in the calibre of blood vessels and the electrophysiological activation of trigeminovascular neurons during a migraine attack [6]. It may also trigger the headache and aura phase of migraine attacks by activating and sensitizing the trigeminovascular system, initiating a series of neural, vascular, and inflammatory events that result in pain [13]. Several lines of evidence for vascular dysfunction have been identified in migraineurs, and there is increasing evidence to suggest that in migraineurs the vascular system is impaired not only within the brain but also in the central retinal artery and posterior ciliary artery [11–14].

Migraine attacks have been reported to be related to decreased blood flow in the optic nerve, retina and choroid [3, 4]. There is increasing evidence that ocular blood flow changes are involved in both the pathogenesis of glaucoma and the progression of glaucomatous damage [15]. Dadaci et al. and Karalezli et al. noted that an increased choroidal thickness during the migraine attack period could reflect an alteration of ocular circulation [16, 17]. However, Dervisogullari et al. and Zengin et al. reported a reduction in choroidal thickness during the migraine attack period in patients with migraine [9, 18]. Demircan et al.proposed that the discrepancy between the results of these studies may be explained by the fact that both groups of migraine evolve from hypoperfusion to hyperperfusion during their time course, although perhaps with a difference in intensity [19]. Although in different studies migraine and its association with retinal and choroidal thickness were reported, to the best of our knowledge, this is the first study to report the effects of migraine on IOP, AL, CCT, ACD, CCR, WtW, PD, and LT.

IOP is dynamic and determined by several variables. The relationship between IOP and these variables can be modeled by the modified Goldmann equation:

$$ \mathrm{I}\mathrm{O}\mathrm{P}=\mathrm{E}\mathrm{V}\mathrm{P} + \left(\mathrm{Q}\ \hbox{--}\ \mathrm{U}\right)/\mathrm{c}, $$

where EVP is the episcleral venous pressure, Q is the aqueous humor flow rate, c is the conventional outflow facility, and U is the pressure-insensitive uveoscleral outflow rate [20].

The ciliary body is a highly vascular tissue, and aqueous humor is produced in the ciliary processes, which form a ring of leaf-like projections into the posterior chamber under the iris root and limbus. Decreased ciliary body blood flow reduces aqueous production, and relatively small changes in vascular tone may result in venous constriction that increases fluid resistance and episcleral venous pressure [21].

Although in our study IOP values slightly increased in migraine patients during pain-attacks, we did not find any significant differences in the mean IOP values between those taken during pain and the headache-free interval (*p* = 0,12) or between migraine patients and healthy participants (*p* = 0,5). Based on different studies that investigated the relationship between ciliary blood flow and aqueous humor production, Kiel et al. proposed that aqueous humor production is independent of ciliary blood flow above a critical level of ciliary perfusion, and blood flow is dependent below that critical level of perfusion [21]. This may indicate that the auto-regulatory mechanisms of the ciliary body perfusion may overcome the decrease of the ciliary body blood flow above that critical level of perfusion in migraine patients.

Our study showed that ACD and LT parameters were increased during migraine attack, whereas the increase was not statistically significant (*p* = 0,4). Several studies showed that changing the ciliary body and choroid thickness might affect ACD by the movement of the iridolenticular diaphragm [22]. Zengin et al. found that choroidal thickness measurements of five patients during an attack showed an acute decrease in choroidal thickness from the values in the same patients during the attack-free period [9]. Ciliary body is the middle part of the uveal tract, and we think that its thickness might be decreased acutely like choroidal thickness during migraine attack. So, thinning of the ciliary body causes the relaxation of the lens zonules and may be one of the important factors associated with the increased lens thickness and increased ACD in migrainous eyes during attack.

In our study, we did not find a statistically significant difference in corneal parameters such as CCR, WtW, and CCT during the headache, headache-free interval, and non-headache control subjects. Kinard et al. compared the structural differences in the sub-basal corneal nerve plexus of chronic migraine patients with a control group. Participants with chronic migraine had a reduced density of the nerve fibers in their cornea, and all migraine subjects had symptoms consistent with a diagnosis of dry eye syndrome. According to the researchers, these changes support the hypothesis that the trigeminal system plays a critical role in the pathogenesis of migraine [23]. Although we did not examine whether dry eye was associated with migraine, dry eye was found to be associated with decreased CCT in previous studies [24]. It was indicated that dry eye might present in migraine patients with greater presence of auras and longer disease and attack durations [25]. The reason we could not detect any change in corneal thickness in patients with migraine may be the low frequency of attacks and short duration of disease.

The pupil has been recognized as a unique organ for the study of the autonomic nervous system [26]. Autonomic nervous system dysfunction in migraine, even if subclinical, can lead to pupillary abnormalities, and it can be observed in migraine patients [27]. Also, an increase in lens thickness might push the iridolenticular diaphragm anteriorly to some extent, which increases pupil diameter slightly [28]. In our study, we were not able to find a significant difference in PD between groups.

Our study had several limitations. First of all, we did not measure ciliary blood flow; however, several studies support our suggestion of decreased blood flow of retrobulber vessels and posterior ciliary artery, according to the vasogenic theory of migraine [14]. Another limitation was related to the effect of the circadian rhythm of aqueous humor flow: aqueous humor flow is normally about 3.0 μl/min in the morning, 2.4 μl/min in the afternoon, and drops to 1.5 μl/min at night, which has been demonstrated in previous studies [29]. However, in our study, we performed the biometer and IOL examinations at the same time of day (10:00–12:00) during the migraine attack and excluded the attack patients out of this time limit, so it was unlikely to be a potential bias in our study. The last major limitation of this study was that the LT measurements had to be performed manually, which remains a potential cause of interobserver bias. To overcome this limitation, the same researcher (YK) performed all measurements.

## Conclusions

No statistically significant difference was detected in IOP and biometric parameters between migrainous during the attack period and the healthy control group. This is a single-center study that has a relatively small sample size; therefore, these findings need to be confirmed in a larger patient group. The impact of migraine on IOP and anterior chamber parameters should be further investigated.

## Abbreviations

ACD, Anterior chamber depth; AL, Axial length; CCR, Corneal curvature radius; CCT, Central corneal thickness; CSD, Cortical spreading depression; IOP, Intraocular pressure; LT, Lens thickness; PD, Pupil diameter; WtW: Corneal diameter.
